# Biosimilar Use and Switching in Belgium: Avenues for Integrated Policymaking

**DOI:** 10.3389/fphar.2022.821616

**Published:** 2022-07-12

**Authors:** Liese Barbier, Steven Simoens, Paul Declerck, Arnold G. Vulto, Isabelle Huys

**Affiliations:** ^1^ Department of Pharmaceutical and Pharmacological Sciences, KU Leuven, Leuven, Belgium; ^2^ Hospital Pharmacy, Erasmus University Medical Center, Rotterdam, Netherlands

**Keywords:** biosimilar, biological, best-value biological, switching, stakeholder incentives, healthcare professional, policy making

## Abstract

**Background:** By improving the affordability and accessibility of biologicals, biosimilar competition provides important benefits to healthcare systems and patients. In Belgium, biosimilar uptake and competition is limited compared to other European markets. Whereas other countries have initiated structured biosimilar introduction or switching plans, no such framework or guiding principles are yet available in Belgium.

**Objective:** This study aims to develop recommendations that can inform policy action in Belgium on biosimilar use, especially in the context of switch decision-making, and this by drawing from the perspectives of healthcare professionals involved in procuring, prescribing, switching and dispensing biologicals including biosimilars.

**Methods:** This study made use of the consensus-building Nominal Group Technique, consisting of a three-step process 1) individual grading, 2) three structured Focus Group Discussions, 3) final individual grading involving an expert group of Belgian healthcare professionals (physician specialists and hospital pharmacists).

**Results:** Participants (*n* = 13) voiced challenges with the use of biosimilars and switching in practice, and a lack of incentives to use them. Six concrete areas for policy development to support stakeholders with biosimilar use and switch decision-making were identified: 1) address stakeholder hesitations regarding (multiple) switching, 2) provide meaningful incentives, 3) guide healthcare professionals with product decision-making, 4), align practical product modalities when possible, 5) involve healthcare professionals in policy making, and 6) provide practical switch support and patient information material, particularly in the ambulatory care setting. For each area, specific consensus-based recommendations were developed. Furthermore, a set of switch management and patient communication principles was derived, including amongst others, generating buy-in from involved stakeholders prior to switching and communicating with a one-voice message.

**Conclusion:** Without cohesive actions to reduce hurdles and without tangible benefits or steering mechanisms, changes in biosimilar use are unlikely in Belgium. To overcome this and stimulate market competitiveness, this study advances a set of concrete policy recommendations. At large, policy makers should develop an integrated policy framework, with a pro-active, best-value biological implementation roadmap that provides guidance and compelling measures to incentivize healthcare professionals to use biosimilars. Particular consideration should go to the ambulatory care setting, since drivers for biosimilar use are quasi absent in this context.

## 1 Introduction

The market entry of biosimilars—biological medicines that are highly similar to an already approved biological medicine (European Medicines Agency, 2014)—has shown to lead to lower treatment costs and in some cases increased patient access to biological therapies (IQVIA, 2020; National Institute for Health and Disability Insurance (NIHDI), 2021a; IQVIA, 2018a), delivering benefits to both healthcare systems and patients. With biological therapies already accounting for 40% of total pharmaceutical spending in Europe and still expanding, biologicals represent a growing budgetary challenge for many European health systems (IQVIA, 2021). Biosimilar medicines are an important lever to manage this growing biopharmaceutical expenditure by fostering competition. However, in order to reap the benefits of biosimilars, healthcare systems must be well organized to allow for effective competition and stimulate their long term presence (IQVIA, 2018b; IQVIA, 2021).

Compared to other European countries, biosimilar market shares are considerably lower in Belgium. In 2020, Belgian biosimilar market shares, with the exception of filgrastim, infliximab and follitropin alfa, continued to be below 20% (Medaxes, 2021). Of the 71 biosimilars (of 16 distinct original biological medicines) that have a valid marketing authorization for the European Union, 34 (of 13 original biological medicines) are available at present (January 2022) in Belgium (BCFI, 2022; European Medicines Agency, 2022) At policy level, awareness exists that changes are required to ensure a more attractive climate for continued biosimilar market presence in Belgium (National Institute for Health and Disability Insurance (NIHDI), 2021b; Beleidscel van de minister van Sociale Zaken en Volksgezondheid, 2016). In 2016, a convention was agreed between the Ministry of Social Affairs and Health with industry and professional associations with the aim of fostering biosimilar uptake (Beleidscel van de minister van Sociale Zaken en Volksgezondheid, 2016). Despite this, and a series of *ad hoc* measures over the past years, biosimilar adoption in Belgium remains challenged (Moorkens et al., 2020a; CM, 2021; Medaxes, 2021; Vandenplas et al., 2021). Notwithstanding the short-term savings that have been realized due to biosimilar market entry (Medaxes, 2021), which can be mainly attributed to the mandatory price reductions that original biologicals undergo in Belgium at the time of biosimilar entry and further confidential discounting in tendering, it can be argued that the way the healthcare system is organized in Belgium may impede the establishment of a sustainable, off-patent biological and biosimilar market environment with continued competition over the longer term (Van Wilder, 2021; Vandenplas et al., 2021). The current system, which is mainly built on generating short-term savings through mandatory price decreases of the originator product, may negate the incentive to opt for a similarly priced biosimilar. While this approach leads to desirable short-term savings for the healthcare system, it may limit the entrance of new biosimilar products down the line and impoverish market competition. Supplementary Table S1 provides an overview of relevant Belgian biosimilar policy parameters.

Policies to ensure a sustainable off-patent biologicals climate, with sustainable competition from biosimilars, are currently missing in Belgium (Dylst et al., 2014; Moorkens et al., 2020a; Vandenplas et al., 2021). In particular, more attention is needed to develop healthcare professional targeted policy measures (Moorkens et al., 2020a). Physicians and pharmacists are key stakeholders as they are the ones in charge of purchasing, prescribing or dispensing off-patent biologicals and biosimilars. Furthermore, specific attention is required to investigate healthcare professional needs and considerations regarding switching and its management as biologics for which biosimilars are available are often used in a chronic treatment setting in which switching may occur.

This study aims to inform policy action for biosimilar use and switch management in Belgium from the perspective of Belgian healthcare professionals, by examining and prioritizing their needs and views in a structured manner.

## 2 Methods

### 2.1 Study Design

**FIGURE 1:**
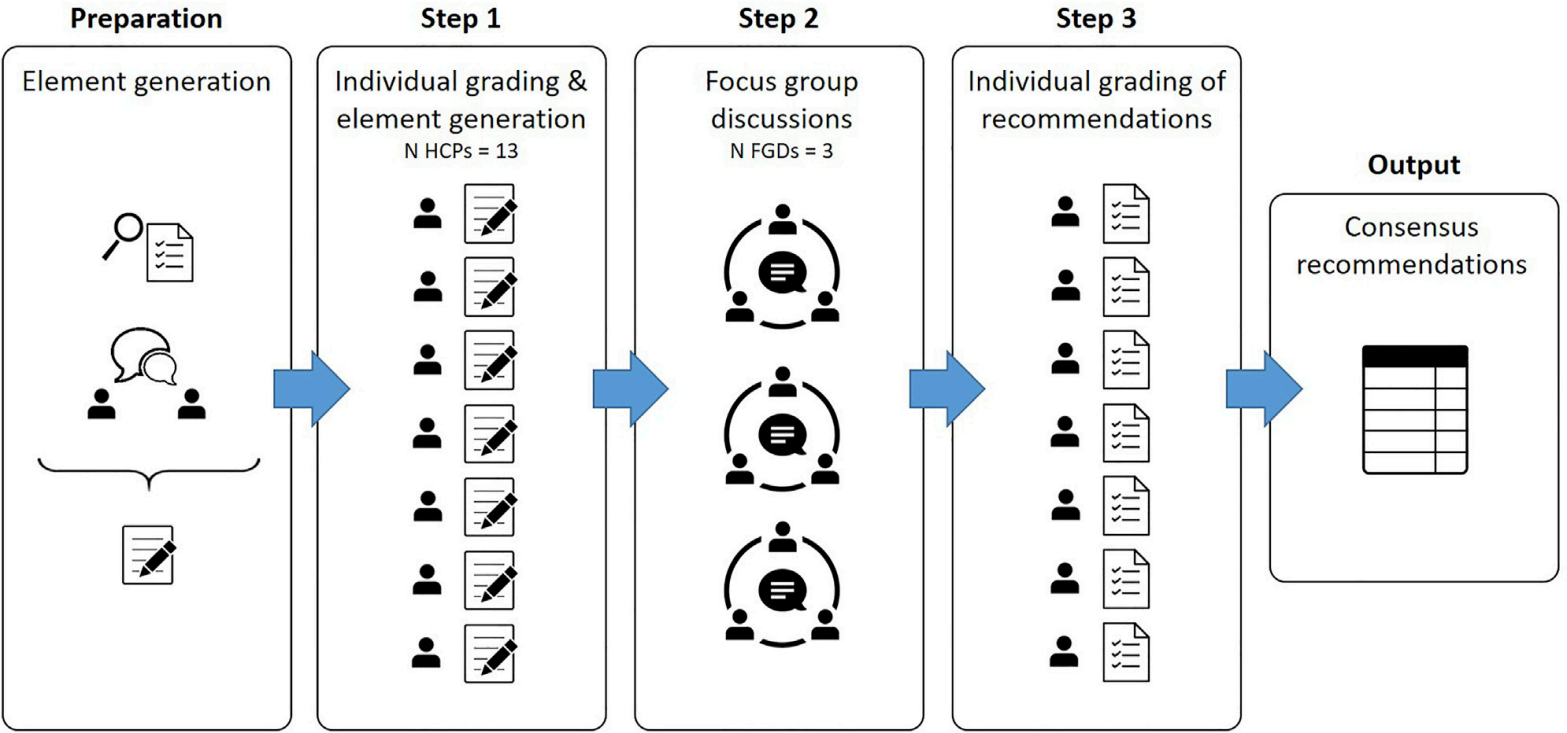
Overview of the applied step-wise Nominal Group Technique. Legend: Figure based on Varga-Atkins et al. (2017) Innovations in Education and Teaching International (Varga-Atkins et al., 2017).

The methodology of the Nominal Group Technique (NGT) was chosen to identify stakeholder priorities and generate recommendations with consensus (McMillan et al., 2016; Varga-Atkins et al., 2017; Manera et al., 2019). The applied NGT methodology consisted of the following steps: 1) initial individual grading and idea generation, 2) three structured group discussions and 3) a second round of individual grading of the derived recommendations. An overview of the different methodological steps is shown in Figure 1.

### 2.2 Setting

The group discussions were organized in person at the University campus Gasthuisberg, Leuven, Belgium, between December 2019 and February 2020. The option to participate online was provided when a participant could not be physically present. Each group discussion lasted approximately two and a half hours. Three researchers were involved in the conduct of the group discussions: a moderator who guided the discussion, and two observers who were in charge of taking notes, drafting summary slides and time keeping.

### 2.3 Participants

Both Belgian physician specialists and hospital pharmacists involved in purchasing, prescribing, switching and dispensing of biologicals, including biosimilars, were purposively selected and invited to participate. Physician specialists across the different disease disciplines in which biosimilars are currently available were invited (dermatology, rheumatology, gastroenterology, oncology, and endocrinology). Participants were invited from both university and regional hospitals across different geographical areas in Belgium. Recruitment was done via e-mail invitation, which included an information sheet with detailed information about the aims and set-up of the study. Upon expression of interest to participate, an informed consent form was shared with the participant. The informed consent form was signed by all participants before participation.

Each group discussion consisted of both physician specialists and hospital pharmacists to capture and stimulate discussion between both stakeholder groups. Each group discussion involved three to six participants (*n* total = 13) (McMillan et al., 2014; McMillan et al., 2016; Manera et al., 2019).

### 2.4 Data Collection and Analysis

#### 2.4.1 Initial Individual Grading

Participants were asked to grade and comment on a number of statements, capturing their individual perspective on topics related to biosimilar use, switch decision-making and management. For this, each participant received a written answer sheet.

The answer sheet consisted of two parts. The first part included questions about the participant’s characteristics. In the second part, participants were asked to indicate their level of agreement with various statements on a 5-point Likert scale (from 1 = strongly disagree to 5 = strongly agree). Statements were categorized in three main themes: 1) possible participant needs regarding biosimilar use and switching, 2) possible elements to consider in biosimilar use and switch decision-making and 3) possible elements to consider when managing a switch in practice. Participants were given the option to provide written comments on any of the statements. Further, an open answer field was included where participants were asked to include additional ideas or points of interest. Topics and statements included in the answer sheet were informed by earlier biosimilar stakeholder research (Barbier et al., 2020a; Barbier et al., 2020b), and participants were asked to add their insights.

Participants’ characteristics and grades from the written answers sheets were analysed descriptively in Microsoft Excel. Each participant was given an identifier code with which the research data were coded (pseudonymized) and subsequently tabulated. The participants’ grades from both grading rounds were analysed descriptively, by calculating mean values for each statement. The participants’ average grading per statement (ranked according to level of importance/agreement) on the first answer sheet are given in Supplementary Tables S2–S4.

#### 2.4.2 Group Discussions

A structured discussion guide was used to inform the group discussions. The guide included an introduction about the role of the moderator and the observers and an explanation about the study. In addition, the guide contained detailed instructions for the researchers to follow throughout the discussion. Each group discussion consisted of several phases. First, the participants were welcomed and an introduction was given. Also the study set-up and aims were explained. In the subsequent discussions, participants were asked to share their needs and perspectives regarding biosimilar use and switch decision-making and management in clinical practice. Participants were offered the opportunity to accentuate the importance given to certain elements, share nuances and discuss their underlying reasoning. The discussion was initiated by inviting each participant to speak. Next, open discussions, structured by the moderator, were held. At the end of the discussion, draft summaries were presented to the participants, on which they were asked to comment and indicate their agreement. A PowerPoint presentation was used to visually guide the session and present discussion elements to the participants.

The structured group discussions were audio-recorded and the audio tapes were transcribed *verbatim*. The transcripts were pseudonymized and qualitatively analysed according to the thematic framework method of Lacey and Luff, which consists of an iterative process of data familiarization, identification of a thematic framework, coding of transcripts with NVivo data analysis software, charting the data by code and mapping and interpretation steps (Lacey and Luff, 2007). Summaries prepared during the group discussions and the written comments from the open answer fields in the answer sheets were tabulated in Microsoft Excel and included for qualitative analysis.

#### 2.4.3 Second Individual Grading

Participants were asked to indicate their level of agreement on a 5-point Likert scale, this time on a concrete set of proposals derived from quantitative analysis of the first individual grading and qualitative analysis of the group discussion transcripts.

For this second and final grading, we considered strong consensus to be achieved for a recommendation when at least 80% of participants agreed with the statement (yes/no) and the overall participant mean level of agreement was ≥4 on the Likert-scale (Kay et al., 2018; Torres et al., 2020). Recommendations with a mean level of agreement ≥3.5 were regarded as recommendations with moderate consensus. If the overall mean level of agreement was <3.5, no consensus was considered to be reached.

## 3 Results

### 3.1 Participant Characteristics

In total, 13 Belgian specialists in rheumatology, oncology, gastroenterology, endocrinology and hospital pharmacy participated across three structured group discussions. In terms of geographical spread, participants worked in either Flanders or Brussels. No healthcare professionals from Wallonia (French speaking Belgian region) participated. Both academic and regional hospitals were represented, with the majority of participants working in an academic setting. A complete overview of the participant characteristics is shown in Table 1.

**TABLE 1:** Participant characteristics.

Characteristics	N participants (*n* total = 13)
Sex	
Female	3
Male	10
Age (years)	
<30	1
30–45	3
>45–60	6
60	3
Years of work experience	
<5	2
≥5—< 10	0
≥10—< 20	2
≥20—< 30	6
≥30	3
Discipline	
Physician specialist	6
Hospital pharmacist	7
Work environment	
Flemish-Brabant	8
Brussels	1
Antwerp	1
West-Flanders	1
East-Flanders	1
Limburg	1
Work environment	
Academic hospital	9
Regional hospital	4
Have you already been involved in decision-making and/or implementation of switching to biosimilars?	
Yes	10
No	3
Have you already encountered multiple switching in practice?	
Yes	3
No	10
Have you already encountered biosimilar-biosimilar switching in practice?	
Yes	1
No	12

N: number.

### 3.2 Overarching Areas for Policy Action With a Set of Consensus Recommendations

**FIGURE 2:**
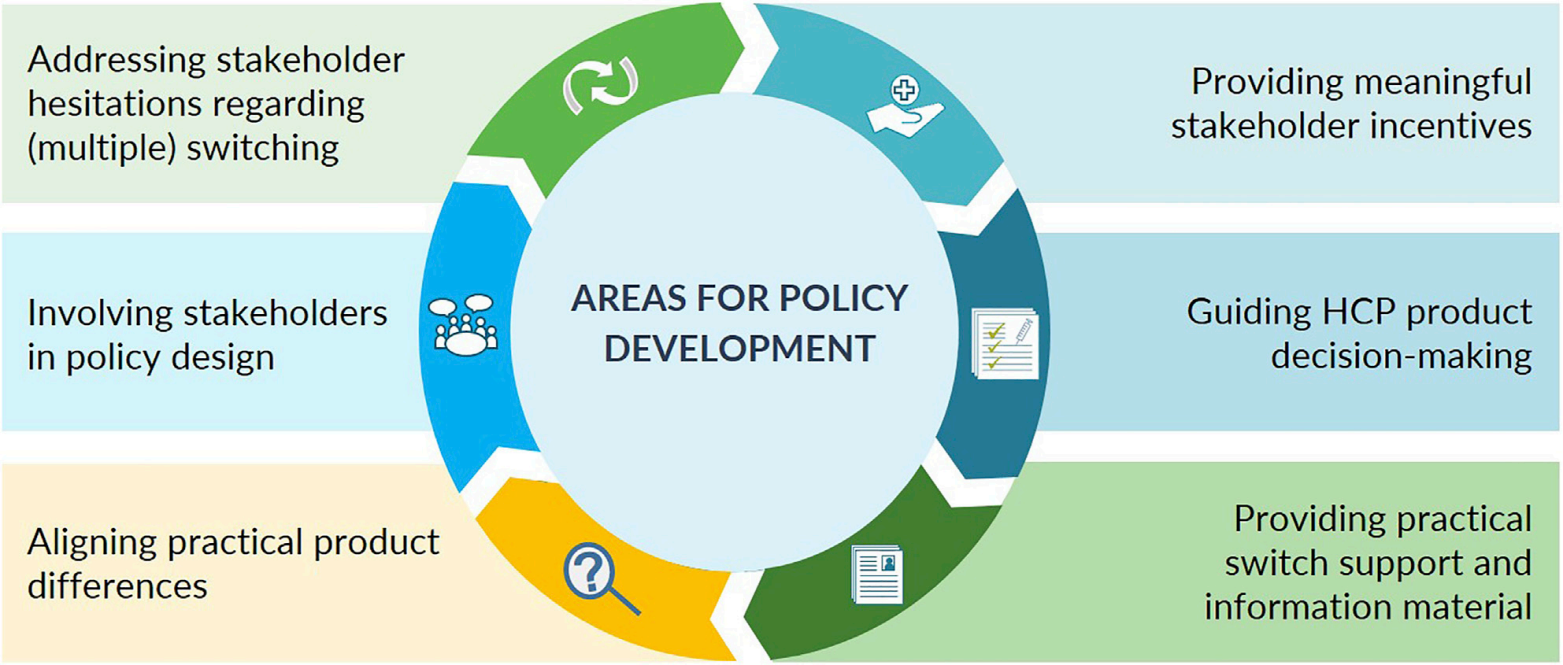
Biosimilar use and switching in Belgium—6 main healthcare professional-identified areas for policy development.

From the analysis, six main areas for policy development regarding biosimilar use and switch decision-making in Belgium emerged: 1) address stakeholder reservations regarding (multiple) switching, 2) provide meaningful stakeholder incentives, 3) guide healthcare professionals with product decision-making, 4), align practical product modalities (to the extent possible), 5) involve healthcare professionals in biosimilar policy making, and 6) provide healthcare professionals with practical switch support and patient information material, particularly in the ambulatory setting. A schematic overview is given in Figure 2. For each of the six main areas for policy development, the context is provided below. The set of concrete recommendations for each area, as derived from participant proposals, together with the level of consensus is shown in Table 2.

**TABLE 2:** Biosimilar use and switch decision-making in Belgium: proposals for policy action from structured healthcare professional group discussions.

	Level of agreement^a^	Level of consensus^b^	Authors’ assessment of priority^c^
**1. Addressing stakeholder hesitations regarding the safety of (multiple) switching**			
*Providing clear and transparent position statements*			
A. The Belgian Federal Agency of Medicines and Health Products should provide a more explicit position on interchangeability, switching and multiple switching between biological reference products and biosimilars. Guidance on the measures that should be taken by healthcare professionals should be more clear. This could help address medico-legal concerns of healthcare professionals when switching	4.0	Strong consensus	High
B. Scientific professional associations should provide position statements and keep these updated about biosimilar use and elements such as switching and interchangeability	4.4	Strong consensus	High
*Pro-actively sharing switch experiences and clinical switch data*			
C. Sharing switch experiences between hospitals can help to generate peer-to-peer guidance and overcome hesitations with biosimilar use	4.1	Strong consensus	High
D. The gathered clinical data regarding switching between biological reference products and biosimilars (sourced from either clinical studies, real world evidence, registries etc.) should be actively communicated (not study per study, but on an aggregated/overview level) to healthcare professionals, instead of made passively available. This will help to ensure that the existing data and information reaches the target audience and can support them with biosimilar use in clinical practice	3.7	Moderate consensus	Intermediate
E. Gathering clinical data about the long-term safety of switching between biological reference products and biosimilars could help to instil further trust about switching among stakeholders. Gathering real world evidence while switching in clinical practice, for example by collecting patient outcomes in a registry or observational study, could be explored to generate long-term safety data	3.6	Moderate consensus	Intermediate
F. Clinical data about multiple switching should be generated, as only limited data are available so far. Gathering real world evidence while switching in clinical practice, by for example organizing an observational study when managing a multiple switch in clinical practice or collecting patient outcomes in a registry, could be a way to generate such multiple switch data	3.9	Moderate consensus	Intermediate
**2. Guiding healthcare professionals with product decision-making: steering cost-effective prescribing and biosimilar use *General***			
*Increasing awareness about prescribing behaviours and increasing transparency*			
A. Awareness among healthcare professionals about cost-effective prescribing should be stimulated	4.4	Strong consensus	High
B. Making prescribing information available on a peer-to-peer level (such as done with the Tool for Administrative Reimbursement Drug Information Sharing (TARDIS) platform for rheumatologists) could allow prescribers to compare own prescribing patterns to these of colleagues on a group level (peer-to-peer benchmarking), and increase awareness about cost-effective prescribing	4.0	Strong consensus	High
C. There should be transparency about financial ties between healthcare professionals and pharma industry (for example by making the beTransparent initiative (https://www.betransparent.be/en/) more well-known or other initiatives that increase transparency)	3.9	Moderate consensus	Intermediate
*Revision of pricing and reimbursement modalities*			
D. Price and reimbursement conditions of same International Non-proprietary Name products with a different route of administration (*e.g.,* subcutaneous and intravenous in the cases of rituximab and trastuzumab) should be reassessed upon biosimilar market entry	4.2	Strong consensus	Intermediate
E. Price and reimbursement conditions of innovator/second-generation products should be reassessed upon biosimilar market entry	3.9	Moderate consensus	Intermediate
*Hospital context*			
*Supporting hospitals with tender organization*			
F. The government should support hospital pharmacies by performing horizon scanning to identify the upcoming loss of exclusivity of reference products and anticipated biosimilar market entry dates	4.2	Strong consensus	High
G. Guidance by responsible bodies should be provided to hospital pharmacists (and procurement colleagues) about the design and application of appropriate tender criteria for off-patent biologics and biosimilars (e.g., *via* a tender template)	3.95	Moderate consensus	High
H. Guidance about the design and application of appropriate tender criteria for off-patent biologics and biosimilars should be provided on an overarching level and allow room for tailoring	3.85	Moderate consensus	High
*Reforming hospital financing*			
I. The reform of the hospital financing system will be important to make hospitals less financially dependent on the revenue generated from discounts in pharmaceutical product procurement	4.7	Strong consensus	Intermediate (high impact, low feasibility)
*Ambulatory care context*			
J. Cost-effective prescribing should be stimulated *via* prescription guidelines/treatment decision trees/steering software. This may help to avoid a shift to prescribing higher priced innovator/second-generation products, which in some cases may have a limited/questionable added clinical benefit compared to the reference product/biosimilar alternatives	3.6	Moderate consensus	High
K. Cost-effective prescribing and biosimilar use should be stimulated via (temporary) prescription quota. The installation of (temporary) prescription quota could be accompanied with a stakeholder incentive	2.6	No consensus	Intermediate
**3. Providing meaningful incentives for involved stakeholders**			
*General*			
A. There should be a transparent reporting about the savings derived from biosimilar use and the allocation	4.4	Strong consensus	Intermediate
*Hospital context*			
B. A gainsharing program, where a part of the tender savings flow back to the clinical unit and healthcare professionals that were involved in the switch (such as for example by financing specialist nurses), should be applied to reward involved healthcare professionals for the time and effort associated with a switch	3.6	Moderate consensus	Intermediate
*Ambulatory care context*			
C. A gainsharing program, where a part of the savings are used for the financing of care processes (budget for a nurse or increased physician consultation honorarium) should be applied to incentivize prescribers in the ambulatory care setting, and reward them for the time and effort associated with a switch	3.8	Moderate consensus	Intermediate
D. An incentive at the level of the patient, by for example lowering patient co-payment for the biosimilar versions, could be a way to stimulate biosimilar use in the ambulatory setting	3.6	Moderate consensus	Intermediate
**4. Aligning practical product differences, to the extent possible**			
A. Product labels (in terms of registered indications) should be aligned between the reference product and its biosimilars	4.8	Strong consensus	Low
B. Reimbursement conditions should be actively and timely aligned between second-generation products, reference product and its biosimilars	4.4	Strong consensus	High
C. Benefits provided in the context of Medical Need Programs, which involved the offering of free goods, should be aligned between reference products and biosimilar medicines	4.5	Strong consensus	Low
**5. Involving healthcare professional stakeholders in policy making**			
A. Biosimilar policy making would benefit from actively involving healthcare professional stakeholders. Involving them in incentive design could for example help to establish incentives that can lead to meaningful improvements in patient care	3.7	Moderate consensus	Intermediate
**6. Providing practical switch support and patient information material, especially in the ambulatory setting/for subcutaneous products**			
A. Practical support (switch management information and resources) should be provided about switching, especially to support stakeholders with subcutaneous (self-administered) product switching/switching in the ambulatory setting	3.9	Moderate consensus	High
B. Independent and objective patient information materials should be prepared to support physicians with switch management	4.0	Strong consensus	High
C. Education and information for healthcare professionals should be extended to community pharmacists and general practitioners, as more and more biosimilars (*e.g.,* for insulin, adalimumab, etanercept) are becoming available in the community pharmacy	4.2	Strong consensus	High

a Participants expressed their level of agreement (LoA) on a five-point Likert scale, with 1 = strongly disagree to 5 = strongly agree. This column shows the calculated mean LoA.

b Strong consensus: when at least 80% of participants agreed with the statement (yes/no) and the mean overall LoA was ≥4 on the Likert scale; moderate consensus: a mean overall LoA of ≥3.5 on the Likert-scale; no consensus: a mean overall LoA of <3.5 on the Likert scale.

c Authors’ assessment of priority is made by considering the following two elements 1) implementation feasibility of the proposal and 2) estimated impact: high, intermediate or low.

#### 3.2.1 Addressing Stakeholder Hesitations Regarding (Multiple) Switching

Participants underlined the reassuring impact that both the availability of supportive data from clinical switch studies and own positive switch experiences have had. Despite the fact that the availability of clinical data regarding switching was considered to be paramount in generating trust in switching, some participants cautioned to continue to request more clinical switch data, which are in essence not a regulatory requirement in Europe (participant quote: “*when will it ever be enough?*”).

While participants largely expressed to be sufficiently assured regarding the safety of a switch from reference product to a biosimilar, they indicated that this was not always the case for their peers. Also concerns were raised by physicians regarding liability in case a switch would lead to an undesirable effect, illustrating that doubts regarding the practice of switching persisted among some of the participants. Especially, questions and hesitations were voiced around switching multiple times between biosimilar and reference product. A few participants mentioned that for this particular reason the tender contract which introduced the first switch to a biosimilar in their hospital was maintained for the maximum duration of 4 years, postponing a second switch as long as possible. Some argued that industry outreach has instilled reservations in the switch debate, accentuating the need for communication from independent bodies. Participants expressed to be comfortable with biosimilar-to-biosimilar switching as long as it would be done in a single switch manner.

While a supportive statement regarding switching from reference products to biosimilars, vice versa or between biosimilars of the same reference product is available from the Belgian medicines agency, the Federal Agency for Medicines and Health Products (FAMHP) (The Belgian Federal Agency for Medicines and Health Products, 2021), some participants requested a more explicit and detailed guiding statement which includes information about multiple switching and guidance on which measures to consider when conducting such a switch. FAMHP mentions that a switch “*must be done with the necessary follow-up.*” Participants mentioned that for them it is not fully clear what is meant with this (routine monitoring as done for the reference product, or a need for additional monitoring). In addition, the suggestion was made for the medicines agency to communicate on available data from clinical switch studies on an aggregated level.

In general, participants pointed out the need for 1) more explicit guidance from regulators and professional stakeholder associations and 2) the collection of multiple switch and/or long-term follow-up switch clinical data, together with a more active leveraging of the available clinical switch data to adequately inform switch decision-making in clinical practice. The recommendations to address stakeholder reservations regarding (multiple) switching, as proposed by participants, are shown in Table 2.

#### 3.2.2 Guiding Healthcare Professional Product Decision-Making

Participants discussed the need for guidance on product decision-making. Besides this being relevant in the context of choosing between biological reference product and biosimilars, participants pointed towards the availability of second-generation and newer therapeutic alternatives, for which the therapeutic benefit is considered equal to the off-patent biologic therapeutic option (reference product and biosimilars). Depending on the context in which product selection takes place, different needs and proposals were formulated by the participants.

In the hospital setting, the tender process can be considered as main driver of product decision-making. Tenders for hospital medicines in Belgium are generally organized by individual hospitals or hospital groups and typically induce competition on the active substance level (*i.e.,* competition on international non-proprietary level) or possibly second-generation products (e.g., long-acting granulocyte colony stimulating factors, pegfilgrastim and lipegfilgrastim are generally grouped in the same lot) (Medicines for Europe, 2021). A first challenge mentioned in the context of hospital tendering pertains to the appropriate use of tender award criteria. As explained by participants, a certain dissonance seems to exist between criteria deemed valid according to healthcare professionals (such as rewarding longer market presence of the product or need for additional switch data) and their competitive nature in terms of the impact they might have on the level playing field. In 2019, the Belgian medicines agency, FAMHP, circulated a letter to Belgian hospitals with information regarding the nature of award criteria, clarifying that these criteria need to be related to the subject matter of the tender itself (The Belgian Federal Agency for Medicines and Health Products, 2019). Regardless, healthcare professionals argued there is still ambiguity regarding the appropriateness of award criteria. As a second challenge, hospital pharmacist participants pointed towards the need for timely information on biosimilar market entry to allow for a timely organization of tenders. As a third point of consideration in the hospital setting, participants underlined the need for a revision of the hospital financing system, as the current set-up incentivizes hospitals to opt for products with high list prices. The price difference between the tendered price and list price is largely retained by the hospital, and accounts for an important portion (approximately 20% on average) of revenue of Belgian hospitals (Belfius, 2020; Barbier et al., 2021). Products with a high list price allow for greatest margin between list price and tendered price and are as such of higher economic value for the hospital (Dylst et al., 2014; Barbier et al., 2021). Finally, also the availability of second-generation biologicals such as subcutaneous formulations for trastuzumab and rituximab were mentioned as reasons why the market opportunity for biosimilars is in some cases limited in hospitals (as currently only the intravenous formulations are available as biosimilar).

For products dispensed by the community pharmacy (i.e., ambulatory setting), product selection was argued to largely remain at the discretion of the individual prescriber. Participants were of the opinion that factors driving product choice may vary between physicians and believed that price is not routinely taken into account. Other factors such as brand loyalty and direct “informal” incentives offered to physicians by pharmaceutical companies were mentioned as potential drivers in decision-making. Moreover, it was emphasized that argumentation to choose for a biosimilar from the perspective of the physician in the ambulatory setting is limited. This point is further discussed under Section 3.2.3. Further, participants argued that physicians may also shift to newer, higher priced products, although the added clinical benefit compared to the off-patent biological for which a biosimilar is available may not always be clearly established. For example, in the treatment of diabetes mellitus with long-acting insulin, product shifts to the higher concentrated insulin glargine formulation Toujeo^®^ [300 U/ml compared to the traditional formulation of 100 U/ml (reference product Lantus^®^ and biosimilar Abasaglar^®^)] or insulin degludec (Tresiba^®^) were mentioned. In rheumatology, physicians mentioned the increasing use of higher priced Janus Kinase Inhibitors, although the clinical benefit of Janus Kinase Inhibitors compared to the off-patent Tumour Necrosis Factor-alfa (TNF) inhibitors (of which several have lost their exclusivity and have biosimilar alternatives on the market: infliximab, etanercept, adalimumab) is questioned. Physician participants pointed out the following elements to potentially explain these shifts to higher priced innovator products: strong pharmaceutical company outreach activities, inclination to possible product innovations (although clear clinical superiority may not always be established) and brand loyalty.

Participants underlined that the availability of newer, competing products should be considered in the context of off-patent biologicals selection making. It was mentioned that shifts to higher priced innovator products should only be made in case clinical superiority is clearly established. Participants argued the need for a reassessment of the value of second-generation products and by extension the entire product class at the time of biosimilar market entry. Although consensus existed among study participants on the importance of cost-effective prescribing, it was argued that the responsibility to consider cost should not be placed on the shoulders of the individual physician. In other words, guidelines and mechanisms were considered needed to assist physicians and steer them to prescribe in a rational way.

In terms of stimulating biosimilar use in the ambulatory care setting, the use of biosimilar market share quota was put forward by some participants during the group discussions. However, no consensus among participants was achieved in the final step of grading for this recommendation. An overview of the proposals on how to support healthcare professionals with product selection making in both the hospital as well as ambulatory care context and steer cost-effective choices are shown in Table 2.

#### 3.2.3 Providing Meaningful Stakeholder Incentives

Participants raised the need for incentives to stimulate biosimilar use and support healthcare professionals with switching in clinical practice. Besides general elements, specific considerations and proposals were given for both the hospital and ambulatory context.

In the ambulatory care setting, participants felt little motivated to prescribe a biosimilar or burden themselves to switch to one due to a perceived lack of benefits for the different parties (i.e., patients, prescribers, payers) involved. Even more so, participants identified hurdles to opt for a biosimilar, especially when it involves switching a patient who is being treated with the reference product. Besides the lack of incentives for the physician to opt for a biosimilar, they argued that also for the patient there is no direct benefit from receiving a biosimilar as it provides similar clinical outcomes compared to the reference product for which (*largely*) the same reimbursement conditions apply [*of note, for some molecules there is in fact a (small) difference in patient co-payment between the originator and biosimilar in Belgium* (BCFI, 2022)]. Furthermore, participants argued that also for the healthcare budget there is no immediate cost advantage in terms of savings as list prices for biosimilars and reference products are largely the same. The general tenor of the participant perspective could be summarized as “*why the hassle of prescribing a biosimilar or switch a patient if there is no benefit for anyone involved?*” Moreover, participating physicians contended that it would require additional consultation time to introduce a biosimilar to the patient, especially if a possibly different injection device would be involved. Moreover, physicians mentioned that unlike in the hospital setting, there is a lack of a framework to support a switch (e.g., staff capacity, information material). This element is further discussed under Section 3.2.6.

The argument that biosimilar suppliers, without the prospect of reaching a meaningful market share, might lose interest in the Belgian market, which in turn might lead to impoverished market dynamics, was perceived as too intangible to consider in daily practice. It was clear from the discussion that structural change needs to be installed in the form of a concrete incentive, benefit or steering mechanism to stimulate biosimilar uptake, if policymakers deem it important to support market plurality and longer-term competition in the ambulatory setting.

The 2019 anti-TNF pilot project, which offered a direct financial incentive to physicians for the prescription of a certain percentage of adalimumab and etanercept biosimilars (National Institute for Health and Disability Insurance (NIHDI), 2021c), was challenged by participants. First, a financial gain on an individual level was perceived as questionable from an ethical point of view. Second, the compensation which was offered was considered insufficient to offset the time investment in terms of patient consultation. Third, the additional administrative practicalities that were associated with the financial incentive pilot were considered to not outweigh the compensation. Participants voiced their support for an incentive which would lead to improvements in patient care. A type of benefit share agreement which would provide funding for additional nurses or pharmacy technicians to assist with the switch process and change in injection device was considered valuable.

In the hospital setting, the lowering of invoicing to 85% for biological medicines for which a biosimilar is available by hospitals to the Belgian national health insurer (National Institute for Health and Disability Insurance, NIHDI) which allows to recoup a part of the savings that are realized in hospital tenders at the national level was recognized as a potent driver for hospitals to organize competitive tenders (National Institute for Health and Disability Insurance (NIHDI), 2021a). As tenders are the main driver of in-hospital product decision making, the need for stakeholder incentives was less pronounced than in the ambulatory setting. Participants argued that tenders do not *perse* result in biosimilar uptake, since also the reference product can win—depending on who offers the most competitive bid. It was argued that a level playing field must be ensured in tenders to secure fair competition between the reference product and biosimilar competitors.

In the hospital setting, incentives on the basis of a benefit share model, where savings are partly reinvested to improve care processes in the department(s) that helped to generate the savings, were considered valuable. Physicians especially stressed the value of a specialist nurse to guide patients with their biological therapy, and the essential role they have in switch management and as such to ensure quality of care. In the tender context, it was debated from which savings bucket such benefit share could come. As the savings are mainly made at the hospital level, a benefit share agreement for tendered products is likely to be negotiated between clinical departments and a hospital’s general management. Alternatively, a benefit share agreement could be made on the basis of the savings generated by the health insurer from the reduced hospital billing (85% billing of the list price for every biological for which a biosimilar alternative is available). However, participants considered the possibility that NIHDI would foresee a benefit share agreement based on the 15% margin to be unlikely, since healthcare budgets are already pressured.

In general, participants mentioned to be not informed about the level of savings that are realized from biosimilar market entry (on the level of the health insurer and hospital) and how these are utilized, expressing a need for more transparency. More transparency was argued to be beneficial to raise awareness among healthcare professionals on the role biosimilars have in creating a more competitive market.

Benchmarking systems, which would enable to mirror own purchasing and prescribing decisions to those of peers, were among the proposed suggestions. An overview of proposals is shown in Table 2.

#### 3.2.4 Aligning Practical Product Modalities, to the Extent Possible

Participants mentioned that in some cases differences between reference biologicals and biosimilars are present in terms of the approved indications, reimbursement conditions and medical need programs. It was mentioned that these “practical” differences may complicate the implementation of biosimilars (also in tenders) and steer choices to the originator product. Arguments were made to align and eliminate these differences where possible, to ensure a level playing field between reference and biosimilar product.

Although the label of biosimilars in terms of registered indications is generally the same as that of their reference product, certain indications might be omitted due to patent or regulatory exclusivity coverage. This may lead to off-label use of the biosimilar in a certain indication and create differences in terms of reimbursement (i.e., no reimbursement for that particular indication).

While generally the reimbursed prices are aligned between the reference product and biosimilar, differences in reimbursement conditions may exist compared to second-generation products. Reference was made to the fact that the higher reimbursed price for lipegfilgrastim, compared to pegfilgrastim (both long-acting granulocyte colony stimulating factors) provided lipegfilgrastim with a competitive advantage in tenders over the pegfilgrastim reference product as well as pegfilgrastim biosimilars. Changes were made to align the reimbursement of pegfilgrastim and lipegfilgrastim in 2020, after which the price of pegfilgrastim products was subsequently lowered again because of a mandatory price reduction, leading again to a competitive advantage for lipegfilgrasim (Vandenplas et al., 2021).

Also Medical Need Programs, which involve the offering of free goods by a pharmaceutical company for a certain disease indication which is still investigated in clinical trials or under evaluation for authorization when there is a medical need for patients, were quoted as a reason to prefer the originator product over the biosimilar in some cases.

While participants considered that these elements may set back the use of biosimilars, it is in practice likely not feasible to align elements of registered indications and Medical Need Programs as they are linked to lifecycle management in terms of seeking approval for new indications that are covered by additional patent/regulatory protection and may involve additional benefits such as the offering of free goods under a Medical Need Program. The concluded overarching area for policy making is thus aligning practical product differences, to the extent this is possible. In terms of aligning reimbursement conditions between reference products, biosimilars and second-generation products, NIHDI should foresee a timely and synchronized revision to eliminate temporary reimbursement differences which negatively impact the level playing field.

#### 3.2.5 Involving Stakeholders in Policymaking

Arguments were made by participants that policy development would benefit from early stakeholder consultation. The anti-TNF financial pilot was given as an example of a policy measure that missed its goal, due to the fact that it was insufficiently aligned with the perspective of the physicians. Involving healthcare professionals in stakeholder-oriented policy measures could help to create a broader support base for these measures, and result in the development of measures that are considered valuable in terms of improving care. Some physicians also felt that opportunities were missed in terms of organizing stakeholder consultations already prior to biosimilar market introduction.

#### 3.2.6 Providing Practical Switch Support and Patient Information Material

Participants mentioned a lack of practical, non-industry sponsored information and guiding principles in relation to switch management. Both materials that can assist with switch management and patient communication were considered needed. In terms of patient communication, reference was made to the 2018 information campaign of the national medicines agency, which offered information brochures and posters. It was pointed out that no specific information on switching was included in these materials (Federal Agency for Medicines and Health Products, 2020a; Federal Agency for Medicines and Health Products, 2020b). Especially in the ambulatory care setting, where there is no structural framework to support healthcare professionals like in the hospital, practical support materials for structured switch management and patient communication are most needed. Where most education initiatives have traditionally focused on specialist physicians and hospital pharmacists, efforts should be expanded to also reach general practitioners and community pharmacists.

### 3.3 Set of Guiding Consensus Principles for Switch Management

In a second part of the study, stakeholders were asked what elements they consider important to take into account when planning a switch. Broad consensus was obtained on a set of guiding principles for switch management, which are outlined in Table 3. Applying a structured switch plan and informing patients was believed to be key, which in turn requires training of staff. In terms of involving patients in the product-decision making, participants argued this to be likely more important for self-administered than IV administered biologicals. Finally, independent information for both healthcare professionals as patients was considered essential to avoid misconceptions.

**TABLE 3:** How to efficiently manage a switch from a biological reference product to a biosimilar or vice versa and inform the patient: guiding principles from structured healthcare professional group discussions.

	Level of agreement^a^	Level of consensus^b^
**When implementing a switch it is important to…**		
*General elements*		
A. Communicate with a one voice principle (coherence in communication and terminology used among physicians, pharmacists, nurses)	4.7	Strong consensus
B. Search for consensus and support from involved stakeholders prior to the switch	4.5	Strong consensus
C. Inform/educate/train involved physicians, pharmacists, nurses about the switch and/or general concepts of biosimilars	4.3	Strong consensus
D. Follow a planned and stepwise approach	3.9	Moderate consensus
*Elements related to the patient*		
A. In the hospital setting: inform patients in advance about the switch	3.7	Moderate consensus
B. For subcutaneous (self-administered) products: inform patients about the switch and involve them in the decision-making	4.5	Strong consensus
C. Provide an opportunity to discuss the switch with the physician/nurse prior to the switch, provide patients with the opportunity to ask questions	4.5	Strong consensus
D. For subcutaneous products (self-administered): provide training to the patient on the new injection device	4.6	Strong consensus
E. Keep it simple. Providing patients with excessive information may invoke uncertainty about the change/biosimilar	4.5	Strong consensus
F. Assess the information need on the individual patient level	4.3	Strong consensus
G. Frame the switch positively, and focus on equality of the treatments	4.7	Strong consensus
H. Allow room for deviation in case a patient objects to switch	3.7	Moderate consensus

a Participants expressed their level of agreement (LoA) on a five-point Likert scale, with 1 = strongly disagree to 5 = strongly agree. This column shows the calculated mean LoA.

b Strong consensus: when at least 80% of participants agreed with the statement (yes/no) and the mean overall LoA was ≥4 on the Likert scale, Moderate consensus: a mean overall LoA of ≥3.5 on the Likert-scale, No consensus: a mean overall LoA of <3.5 on the Likert scale.

## 4 Discussion

Fifteen years after the first biosimilar approval in Europe, Belgium continues to lag behind in terms of biosimilar market competition compared to other European countries. With the aim of creating sustainable competition in the off-patent biological medicines market in Belgium to realize much needed healthcare system benefits (savings and optimal patient treatment access), it is essential to stimulate the entry and use of biosimilar medicines. This study presents a structured examination and a concrete set of policy recommendations to inform biosimilar policy making and switch management in Belgium.

### 4.1 Physician Motivation to Use Biosimilars

Whereas findings showed that participants were overall confident to use and switch to biosimilars, a clear lack of motivation and incentives for healthcare professionals to choose for a biosimilar in practice was unveiled. Especially in the ambulatory setting, opting for a biosimilar was considered to be of limited purpose and burdensome. Because of the mandatory price reduction off-patent biologicals undergo when a biosimilar alternative enters the market, differences in list price between reference biologicals and biosimilars tend to be small, negating a direct, visible reason to opt for the biosimilar alternative (National Institute for Health and Disability Insurance (NIHDI), 2021d). While the system of mandatory price decreases in Belgium locks in substantial savings for the national health insurer, it limits biosimilar uptake and neutralizes for healthcare professionals the reason to actively choose for a biosimilar. Whereas in the hospital context, tendering drives biological selection making and competition beyond the list price level, no such driver exists for products dispensed via the community pharmacy. In this context, product selection is up to the individual prescriber. Because of the generally small price differences between reference product and biosimilar in Belgium and general perceived lack of benefit, physicians are little motivated to prescribe a biosimilar or invest time in switching a patient.

Despite the fact that differences in list price between reference products and biosimilars tend to be small, a 50% adoption of the biosimilars adalimumab and etanercept would still lead to yearly savings for the national health care budget of approximately €1,5 million and €4 million, respectively (Vandenplas et al., 2020). To capture these short term budgetary savings, and create a more attractive biosimilar market environment over the longer term, clear push and pull mechanisms need to be installed. Besides the 2019 financial incentive pilot, which offered a financial bonus at the level of the individual prescriber to prescribe etanercept and adalimumab biosimilars (both dispensed in the ambulatory setting in Belgium) (National Institute for Health and Disability Insurance (NIHDI), 2021c), no healthcare professional incentives have been rolled out. To change the status quo of biosimilar use in ambulatory care, more compelling measures under the form of temporary market share quota for biosimilars are required. While for the latter, no consensus was reached in this study among healthcare professionals—likely explained by the fact that such measure touches upon physician prescribing autonomy—biosimilar quota have been implemented with success in neighbouring countries and are arguably indispensable to drive change. While biosimilars are currently part of the quota for prescribing of “cheap” medicines, this measure is not effective in stimulating biosimilar use in its current form, since both reference and biosimilar medicines fall in the category “cheap” medicines due to the mandatory price decrease system (National Institute for Health and Disability Insurance (NIHDI), 2021e).

Although prescribers are generally little motivated to consider a biosimilar or a switch in a non-tender driven setting—they appear to be open to shift towards newer versions of existing products, second-generation products and new therapeutic alternatives. This finding was confirmed by a recent analysis of Belgian market data, demonstrating such shifts after loss of exclusivities of originator biologicals (Vandenplas et al., 2021). In some cases the therapeutic added value of these newer and often more expensive alternatives has not been clearly established compared to the off-patent originator biological and biosimilar, especially relative to its higher price (Gossec et al., 2016; Smolen et al., 2020; Torres et al., 2020). Physicians’ brand loyalty, low price sensitivity and the “promise” attached to innovation were identified in this study as drivers that could explain this shift in prescribing behaviour. While physicians have a societal responsibility to prescribe in a cost-effective way, appropriate systems should be in place to support this in every day practice (e.g., software with preferred product ranked first). Mandatory price decreases and biosimilar market entries alter the cost-effectiveness of a biological therapy, and should as such trigger a revision of reimbursement modalities within the broader therapeutic class and even other competing product classes (which, e.g., may result in alignment of reimbursement modalities or changes in treatment line) (Moorkens et al., 2020b; Simoens and Vulto, 2021). This should in turn be reflected in prescribing guidelines and prescribing software in order to offer prescribers a framework for cost-effective prescribing.

### 4.2 Tailored and Integrated Policymaking

The study findings highlight the need for a tailored and integrated approach when it comes to installing policy measures for biosimilar medicines. Instead of installing *ad hoc* and short-term cost containment measures, Belgium requires a structured policy framework consisting of integrated measures that foster a long-term healthy competitive climate (Moorkens et al., 2020a; Vandenplas et al., 2021). As exemplified by the set of recommendations derived in this study, there is no one silver bullet solution. Healthcare professionals require policy action on multiple dimensions. While policy makers should strive for a holistic framework, a prioritization can be helpful when installing policy measures in practice. Based on the estimated feasibility of the recommendations and their estimated impact, we assigned recommendations a high, intermediate or low priority (Table 2).

In addition, policy measures should be tailored to the appropriate dispensing (hospital versus ambulatory) and product context (IV versus self-injectable, disease area/specialty). Switching a subcutaneous administered biological may require more time from the involved healthcare professionals compared to intravenous administered products, due to possible differences in injection devices. Also the dispensing context requires specific consideration as the incentive and decision-making structure for in-hospital and community pharmacy dispensed biologicals are distinct as explained above.

**FIGURE 3:**
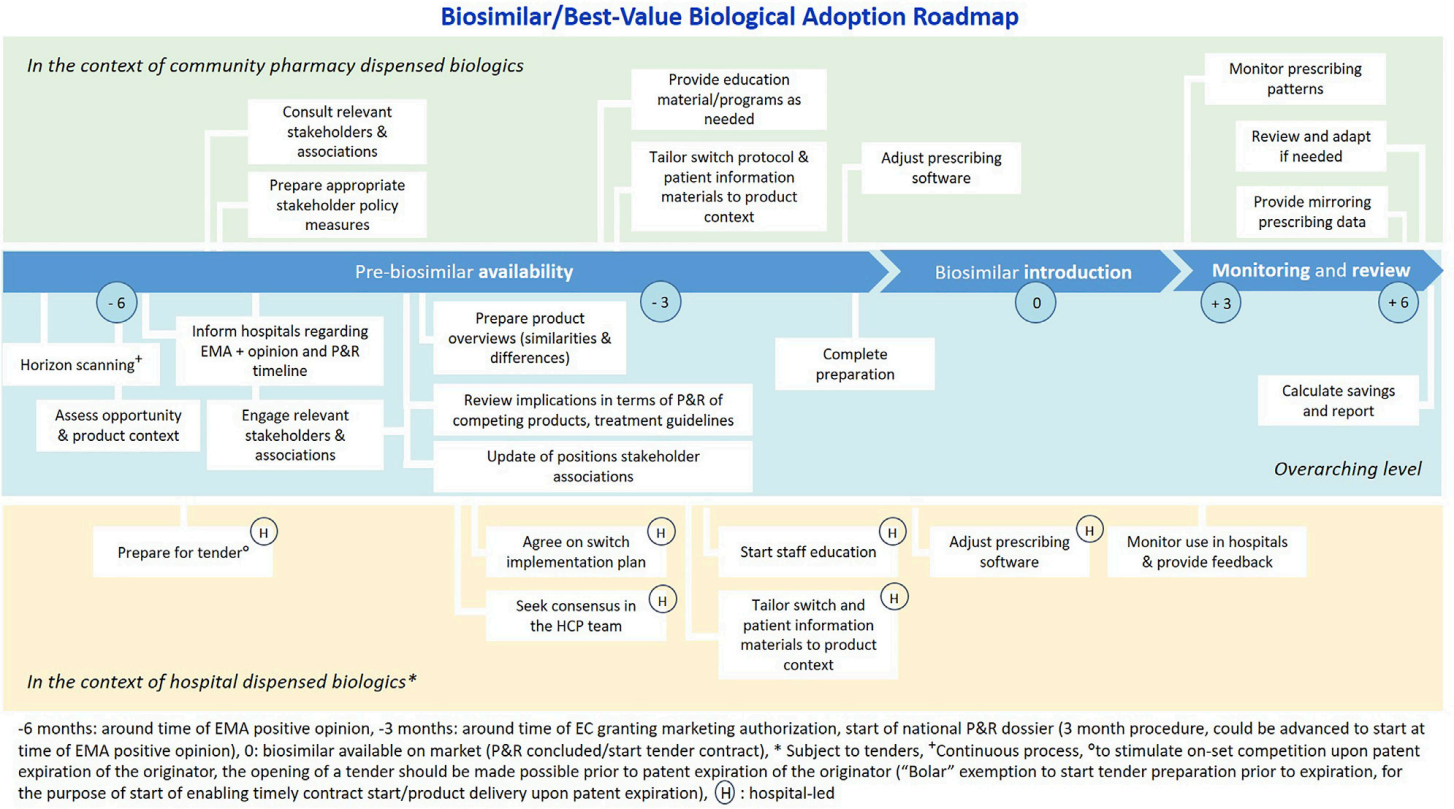
Biosimilar/Best-Value Biological Adoption Roadmap, relevant for the Belgian context. Legend: Figure developed based on the Cancer Vanguard NHS Biosimilar Adoption Process Timeline (The Cancer Vanguard Project, 2017), applied and tailored to the Belgian context

A proposed Biosimilar/Best-Value Biological Adoption Roadmap is shown in Figure 3. Timely preparation and early stakeholder engagement are essential to anticipate to challenges and stimulate on-set competition. In addition, it also gives a clear signal to involved stakeholders; industry and healthcare professionals, about the envisioned role that currently available and incoming biosimilars have. In Ireland, the UK and Denmark best-practice examples of pro-active biosimilar/best-value biological implementation frameworks are in place (The Cancer Vanguard, 2020; Health Service Executive Ireland (HSE), 2021; The Cancer Vanguard Project, 2017; Duggan et al., 2021; Jensen et al., 2020; Bartels and Behnk, 2019; NHS England, 2017). The term best-value biological is preferred to emphasize that rather than high biosimilar uptake, the goal is to achieve healthy competition and sustainable market dynamics that ensure affordable patient access.

### 4.3 Switch Management

While the increasing use of biosimilars in clinical practice and growing body of clinical data regarding switching was argued to have led to increased stakeholder confidence in biosimilars, some stakeholder uncertainties appear to remain, particularly in the context of multiple switching. Hurdles associated with switching in terms of stakeholder uncertainty and practical feasibility should be mitigated for. The following actions are advanced:

1) Develop managed biosimilar introduction and switching protocols with patient communication strategies to provide a clear and structured framework to assist healthcare professionals with biosimilar use and switch management. Besides building trust and practically supporting healthcare professionals, a managed switch program with appropriate patient communication strategies may be a successful way to counter the nocebo effect which has shown to lead to higher discontinuation rates among patients (Tweehuysen et al., 2016; Razanskaite et al., 2017; Avouac et al., 2018; Scherlinger et al., 2018; Schmitz et al., 2018; Tweehuysen et al., 2018; Barbier et al., 2020c; Haghnejad et al., 2020). Consensus was reached in this study on a set of guiding switch principles, which may shape such a managed switch protocol and add to previously published switch management protocols and guidance materials in other European countries (The Cancer Vanguard, 2020; Health Service Executive Ireland (HSE), 2021; NVZA, 2017; ESNO, 2018).
2) The available clinical data from switch studies should be, together with sharing of peer-to-peer experiences, leveraged in an aggregated and active way to inform healthcare professionals. Clinical outcomes of switching between reference products and biosimilars, and increasingly of multiple switching, have been reported for several presently available products in a large number of scientific publications (Cohen et al., 2018; Barbier et al., 2020c). Data from both randomized controlled trials as well as from observational studies did not corroborate any of the voiced safety concerns with switching (Cohen et al., 2018; Barbier et al., 2020c).
3) Regulators should provide a more explicit and homogenous scientific position regarding biosimilar interchangeability and (multiple) switching (Barbier and Vulto, 2021; Barbier et al., 2022). Similarly, clear and up-to-date position statements from learned healthcare professional and patient associations regarding biosimilar use and switching are paramount in building trust among healthcare professionals (Somers et al., 2020).

### 4.4 Study Strengths and Limitations

The following study strengths and limitations are relevant to take into account. The Nominal Group Technique is a recognized consensus method to identify priorities of stakeholders and develop recommendations for integration in healthcare policy making (Varga-Atkins et al., 2017; Manera et al., 2019). Compared to individual stakeholder interviews, the NGT stimulates participants to exchange views on a group level, which consequently allows deepening the discussion with input from different viewpoints. Compared to standard focus group discussions, which also stimulate the exchange of views, the NGT enables a more balanced contribution from and consideration of views of all study participants because of the individual grading steps (two rounds of written feedback) and structured discussion approach by calling pro-actively upon each person to provide feedback (Potter et al., 2004; McMillan et al., 2016).

A purposive sample of stakeholders with relevant expertise was invited to generate a range of ideas and solutions regarding the study topic (Potter et al., 2004; Manera et al., 2019). A heterogeneous participant sample was assembled, with the purpose of reflecting the considerations of the broader spectrum of healthcare professionals who are exposed to biosimilars. The interaction triggered between healthcare professionals representing different medical specialities, dispensing contexts and regional and academic hospitals allowed to formulate nuanced recommendations and underlined the importance of tailored approaches to the product and dispensing context. Consequently, it became clear that policy measures should strive beyond a one-size fits all policy approach and tailor to these specific needs which the study design allowed to differentiate for. Although participants with a diversity in therapeutic and dispensing context where invited, it is worth mentioning that no healthcare professionals from the French-speaking region of Southern Belgium, Wallonia, participated. Where general challenges are expected to be similar across Belgium, regional differences in healthcare professional attitudes may exist. Earlier research reported that the knowledge about biosimilars may be higher among Flemish physicians compared with Wallonian colleagues. As such, the need for information and guidance as part of integrated policy action may be even higher in Wallonia. As with qualitative research in general, study results are bound to the participant sample and study context. While this study focused on biosimilar use and switching in the Belgian context, the findings and proposed strategies may be transferable to other EU countries and jurisdictions that face similar challenges, albeit taking into account the intricacies and financing structures of the respective healthcare system.

## 5 Conclusion

In conclusion, this study shows that healthcare professionals experience challenges with biosimilar use and switch management in Belgium. Challenges are largely attributed to a lack of guidance and tangible benefits or steering mechanisms for healthcare professionals to use them, and particularly so for biosimilars dispensed in the ambulatory care. In order to mature into a competitive and long-term sustainable off-patent biological medicines climate, Belgian policy makers should strive for an integrated policy framework, with a clear, best-value biological implementation roadmap with compelling measures to incentivize biosimilar use and support healthcare professionals with switch management.

## Data Availability

The datasets presented in this article are not readily available because they contain information that could compromise participants’ privacy and consent. Requests to access the datasets should be directed to liese.barbier@kuleuven.be.
