# Supplementary material for: Biosimilar Use and Switching in Belgium: Avenues for Integrated Policymaking
Source: Front Pharmacol. 2022 Jul 12;13:821616. doi: 10.3389/fphar.2022.821616 (PMC9315422; doi:10.3389/fphar.2022.821616)
Supplement: Supplementary file 1 [file Table1.DOCX]

Supplementary Material

**Biosimilar use and switching in Belgium: avenues for integrated policymaking**

**Liese Barbier^1^, Steven Simoens^1*^, Paul Declerck^1^, Arnold G. Vulto^1,2+^, Isabelle Huys^1+^**

^1^KU Leuven, Department of Pharmaceutical and Pharmacological Sciences, Herestraat 49 box 521, 3000 Leuven, Belgium

^2^Hospital Pharmacy, Erasmus University Medical Center, box 2040, 3000 CA Rotterdam, the Netherlands

*** Correspondence:**Liese Barbier
[liese.barbier@kuleuven.be](mailto:liese.barbier@kuleuven.be)

| Table S1. The Belgian biosimilar policy environment | |
| --- | --- |
| Biosimilar market shares | Market shares vary across product types. For most biosimilars, market shares in 2020 were below 20% (1),(2).* |
| Pricing | List prices are negotiated on a case-by-case basis, and cannot exceed the reference product’s list price (3). Original biologicals undergo mandatory price reductions at the 12-year reimbursement mark - if no longer protected by patent or other exclusivities at that time - which can be up to 47,18% depending on yearly product turnover. If biosimilar market entry proceeds the 12-year mark of the original biological, the mandatory price reduction is expedited (4). On list price level, generally only limited differences exist between the reference product and its biosimilar(s) (2),(5). |
| Dispensing context | In public pharmacies, medicines are dispensed at the publicly available list price. For hospital-dispensed biologicals, which are subject to tenders, confidential discounts are offered. The difference between the net tendered price and list price is largely retained by the hospitals (reimbursement limit for biologicals for which a biosimilar is authorized has been reduced by the national health insurer (NIHDI: National Institute for Health and Disability Insurance) to 85%) (6). |
| Pricing & reimbursement procedure | Takes 90 days (7),(8). Evaluation for reimbursement is conducted by the Commission for Reimbursement of Medicinal Products, and the final decision is taken by the Minister of Social Affairs and Health. The Minister of Economic Affairs is in charge of setting the maximum price (8). |
| Interchangeability | The Belgian Federal Agency of Medicines and Health Products (FAMHP), has no publicly available statement on interchangeability of biosimilars with their reference product (9). |
| Switching | Switching can be done under the responsibility of the prescribing physician. FAMHP advises that a switch must be accompanied with the necessary follow-up and accurate recording of the modification. Excluding International Non-proprietary Name prescribing for biologicals is recommended by FAMHP, to avoid switches without follow-up by the prescriber (9). |
| Substitution | Substitution by the pharmacist without consulting the prescriber is not allowed in Belgium for biological medicines (9). |
| Physician incentives | In the ambulatory care setting: a temporary pilot project in 2019 introduced a personal financial incentive for physicians who prescribed a certain percentage of adalimumab and etanercept biosimilars. Depending on the percentage of biosimilar prescribing (5, 10 or 20%), physicians could receive €750, €1000 or €1500 (10).  In the hospitals: benefit-sharing agreements, in which savings from tenders partly flow back to the hospital department, are generally not installed (11). |
| National education & info | FAMHP and NIHDI launched end of 2018 a national information campaign with dedicated website, radio commercials, brochures and posters (12). |
| *FAMHP: Federal Agency of Medicines and Health Products, NIHDI: National Institute for Health and Disability Insurance*  **Exceptions include infliximab, filgrastim and follitropin alfa for which biosimilars attained respectively approximately 39%, 34%, 46% market share in 2020* (1)*.* | |

1. Medaxes. Facts & Figures - Biosimilar medicines in Belgium: unmet potential. 2021.

2. Moorkens E, Vulto AG, Huys I, Vulto AG. Biosimilars in Belgium : a proposal for a more competitive market. Acta Clin Belg. 2020;May(13):1–12.

3. Belgisch Staatsblad. Koninklijk besluit tot vaststelling van de procedures, termijnen en voorwaarden inzake de tegemoetkoming van de verplichte verzekering voor geneeskundige verzorging en uitkeringen in de kosten van farmaceutische specialiteiten (1 FEBRUARI 2018) [Internet]. 2018. Available from: https://www.ejustice.just.fgov.be/cgi_loi/change_lg.pl?language=nl&la=N&cn=2018020122&table_name=wet

4. National Institute for Health and Disability Insurance (NIHDI). De prijsdalingsmaatregel voor de biologische geneesmiddelen [Internet]. [cited 2021 Jun 15]. Available from: https://www.inami.fgov.be/nl/themas/kost-terugbetaling/door-ziekenfonds/geneesmiddel-gezondheidsproduct/terugbetalen/specialiteiten/Paginas/prijsdalingsmaatregel-biologische-geneesmiddelen.aspx#Aanvullende_maatregel:_de_

5. BCFI. BCFI medicines repertorium [Internet]. [cited 2022 Jan 18]. Available from: https://www.bcfi.be/nl/chapters

6. National Institute for Health and Disability Insurance (NIHDI). Terugbetaling van geneesmiddelen: wat is gewijzigd sinds 1 april 2019 [Internet]. [cited 2021 Jun 10]. Available from: https://www.inami.fgov.be/nl/professionals/andere-professionals/farmaceutische-industrie/Paginas/terugbetaling-geneesmiddelen-01042019.aspx#Daling_tot_85%25_voor_de_facturatie_van_bepaalde_geneesmiddelen_in_het_ziekenhuis

7. Van Wilder P. The Off-Patent Biological Market in Belgium : Is the Health System Creating a Hurdle to Fair Market Competition? Pharmaceuticals. 2021;14(352).

8. Wyckmans P, D’herde M. Pricing & Reimbursement 2020 Belgium [Internet]. [cited 2021 Jun 15]. Available from: https://www.globallegalinsights.com/practice-areas/pricing-and-reimbursement-laws-and-regulations/belgium

9. FAMHP. Biosimilars [Internet]. [cited 2021 May 11]. Available from: https://www.famhp.be/en/human_use/medicines/medicines/MA_procedures/types/Biosimilars

10. National Institute for Health and Disability Insurance (NIHDI). Biosimilaire geneesmiddelen: incentive voor het voorschrijven van biosimilaire geneesmiddelen buiten het ziekenhuis [Internet]. [cited 2021 Jun 15]. Available from: https://www.inami.fgov.be/nl/themas/kost-terugbetaling/door-ziekenfonds/geneesmiddel-gezondheidsproduct/geneesmiddel-voorschrijven/Paginas/biosimilaire-geneesmiddelen-buiten-ziekenhuis.aspx

11. Barcina Lacosta T. Gainsharing programs as a tool for the cost-effective prescribing of biologics: An analysis of implementation challenges across Europe. In: Global Implementation Conference 2021 Virtual storyboard presentation [Internet]. 2021. Available from: https://gic.globalimplementation.org/storyboards/

12. Federal Agency for Medicines and Health Products. Nieuwe campagne om patiënten te informeren over biologische geneesmiddelen en om het voorschrijven van biosimilaire geneesmiddelen aan te moedigen [Internet]. [cited 2020 Nov 22]. Available from: https://www.fagg.be/nl/news/nieuwe_campagne_om_patienten_te_informeren_over_biologische_geneesmiddelen_en_om_het

| **Table S2. Possible needs regarding switching -** ­­­­­­­­­­­­Initial individual grading | |
| --- | --- |
| Grading scale: 1= strongly disagree, 2= disagree, 3= neutral, 4= agree, 5= strongly agree  1 – <2, 2 – <3, 3 – <4, 4 – 5 | |
|  | Average grade |
| **Evidence supporting switching** | |
| - There is a need for further evidence generation about multiple switching (alternating) | 3,3 |
| - There is a need for further evidence generation about biosimilar to biosimilar switching | 3,1 |
| - There is a need for further evidence generation about the potential nocebo effect when switching | 3,1 |
| - There is a need for further evidence generation about switching | 2,6 |
| **Guidance and information** | |
| - There is a need for (more or clear) guidelines from scientific associations about switching and interchangeability | 3,8 |
| - There is a need for more guidance on how to implement a switch | 3,6 |
| - There is a need for guidance/measures about how to circumvent the potential nocebo effect when switching | 3,5 |
| - There is a need for (more or clear) guidelines from the Federal Agency for Medicines and Health Products about switching and interchangeability | 3,5 |
| - There is a need for (more or clear) guidelines about switching and interchangeability on European regulatory level | 3,4 |
| **Information about the potential time/cost associated with switching** | |
| - There is a need for information about the potential costs associated with the implementation of a switch | 3,3 |
| - There is a need for information about the time spent to implement a switch | 3,0 |
| **Support when implementing a switch** | |
| - There is a need for education of involved stakeholders to effectively implement a switch | 3,8 |
| - There is a need for remuneration or other types of incentives to compensate for the potential time/cost associated with switching | 3,3 |
| - There is a need for technical support/extra staff to help implementing a switch | 3,0 |

| **Table S3. Importance given to possible elements to consider when deciding to switch -** ­­­­­­­­­­­­Initial individual grading | |
| --- | --- |
| Grading scale: 1= not at all important, 2= not important, 3= neutral, 4= important, 5= very important  1 – <2, 2 – <3, 3 – <4, 4 – 5 | |
|  | Average grade |
| When deciding on the implementation of a switch it is important to… | |
| - Take the (potential) difference in injection material into account (relevant for subcutaneous products) | 4,0 |
| - Take the clinical data about switching into account | 3,8 |
| - Take the (potential) difference in formulation (e.g. citrate free formulation of adalimumab) into account | 3,6 |
| - Take the (potential) cost associated with switch implementation into account | 3,5 |
| - Take the (potential) time associated with switch implementation into account | 3,0 |
| - Take the switch support provided by the supplier into account | 2,9 |

| **Table S4. Importance given to possible elements to consider when implementing a switch –** Initial individual grading | |
| --- | --- |
| Grading scale: 1= not at all important, 2= not important, 3= neutral, 4= important, 5= very important  1 – <2, 2 – <3, 3 – <4, 4 – 5 | |
|  | Average grade |
| When implementing a switch it is important to… | |
| **General elements** | |
| - Communicate with a one voice principle (coherence in communication and terminology used among physicians, pharmacists, nurses) | 4,6 |
| - Search for consensus and support from involved stakeholders prior to the switch | 4,6 |
| - Inform/educate/train involved physicians, pharmacists, nurses about the switch and/or general concepts of biosimilars | 4,4 |
| - Follow a planned and stepwise approach | 4,0 |
| - Take measures to improve product traceability | 3,6 |
| - Monitor trough levels and/or anti-drug antibodies | 3,5 |
| **Elements related to the patient** | |
| - Provide training to the patient on the new injection device | 4,5 |
| - Provide an opportunity to discuss the switch with the physician/specialist nurse prior to the switch | 4,5 |
| - Frame the switch positively, by focusing on equality of the treatments, using positive descriptions to avoid negative expectations | 4,5 |
| - Provide information on why the patient is being switched | 4,2 |
| - Assess the information need on an individual patient level | 4,2 |
| - Provide information about the concept of biosimilars | 3,8 |
| - Involve and consult the patient in the decision making | 3,7 |
| - Offer the opportunity to the patient to switch back to the reference product in case of any experienced issues | 3,5 |
